# The Emerging Role of Phosphodiesterase 5 Inhibition in Neurological Disorders: The State of the Art

**DOI:** 10.3390/cells13201720

**Published:** 2024-10-17

**Authors:** Clara Crescioli, Maria Paola Paronetto

**Affiliations:** 1 Department of Movement, Human and Health Sciences, University of Rome Foro Italico, Piazza Lauro de Bosis, 6, 00135 Rome, Italy; 2 Laboratory of Molecular and Cellular Neurobiology, Fondazione Santa Lucia IRCCS, Via del Fosso di Fiorano, 64, 00143 Rome, Italy

**Keywords:** neuroinflammation, neurodegeneration, PDE5i, cGMP, sildenafil

## Abstract

Growing evidence suggests that neuroinflammation is not just a consequence of neurodegeneration in pathologies such as Alzheimer’s disease, Parkinson’s disease, Huntington’s disease or Amyotrophic lateral sclerosis, but it is rather a determinant factor, which plays a pivotal role in the onset and progression of these disorders. Neuroinflammation can affect cells and processes in the central nervous system (CNS) as well as immune cells, and might precede protein aggregation, which is a hallmark of the neurodegenerative process. Standard treatment methods are far from being able to counteract inflammation and delay neurodegeneration. Remarkably, phosphodiesterase 5 inhibitors (PDE5is), which represent potent vasoactive drugs used as a first-line treatment for erectile dysfunction (ED), display important anti-inflammatory effects through cyclic guanosine monophosphate (cGMP) level stabilization. Since PDE5 hydrolyzes cGMP, several studies positioned PDE5 as a therapeutic target, and more specifically, PDE5is as potential alternative strategies for the treatment of a variety of neurological disorders. Indeed, PDE5is can limit neuroinflammation and enhance synaptic plasticity, with beneficial effects on cognitive function and memory. The aim of this review is to provide an overview of some of the main processes underlying neuroinflammation and neurodegeneration which may be potential targets for PDE5is, focusing on sildenafil, the most extensively studied. Current strategies using PDEis for the treatment of neurodegenerative diseases will be summarized.

## 1. Introduction

Phosphodiesterases (PDEs) represent a group of enzymes controlling many intracellular signals connected with the second messengers cyclic adenosine monophosphate (cAMP) and cyclic guanosine monophosphate (cGMP) [1].

PDEs are expressed in all human tissues, and are sub-grouped into 11 subfamilies which share the main catalytic function of hydrolyzing the 3′ cyclic phosphate bond of either cAMP or cGMP, or both, but display different substrate specificities and intracellular localization [1,2]. From the hydrolytic breakdown of cyclic adenosine monophosphate (cAMP) and cyclic guanosine monophosphate (cGMP) into the biologically inactive derivates 5′-AMP and 5′-GMP, respectively, PDEs can regulate many biological signals and functions. Mammalian PDEs include distinct PDE isoforms generated through alternative pre-mRNA splicing, multiple promoter usage and alternative transcription start sites in humans, rats and mice.

PDEs are divided into three main categories: PDE4, PDE7 PDE8 which are specific to cAMP hydrolysis; PDE5, PDE6 and PDE9 which are specific for cGMP hydrolysis; and PDE1, PDE2, PDE3, PDE10 and PDE11 which have dual specificity for cAMP and cGMP. Although exhibiting different affinities, PDEs share a common structural organization, with a conserved catalytic core of approximately 270 amino acids. The structural determinants of isoform-dependent subcellular localization and specific interactions are located within the PDE enzyme N-terminal region, which can be post-translationally modified to regulate both enzymatic activity and localization in response to external and internal stimuli. Furthermore, the N-terminal domains of PDE1, 4 and 5 also include dimerization domains and autoinhibitory modules [3,4].

Thus, PDEs play a pivotal role in the regulation of many cellular functions because of their ability to control intracellular levels of cAMP/cGMP together with the actions of the adenylyl and guanylyl cyclases responsible for the synthesis of cAMP and cGMP. Interestingly, beside the regulation of cyclic nucleotide content within the cells, PDEs can drive single-cell responses to intra- and extracellular signals, establishing subcellular compartmentalization in nanodomains or individual pockets for cyclic nucleotide signaling [3]. A variety of factors, including tissue type, health/disease condition or aging, can affect PDE location; this aspect should be further evaluated, especially when considering PDEs as potential therapeutic targets [3,4,5,6,7,8,9,10]. Indeed, many companies have exploited the distinct trait of PDEs to interfere with and regulate cell signaling, developing selective drugs targeting specific PDE types [11]. In fact, PDE dysfunction has been found to be associated with or even precede several human diseases, such as cardiovascular diseases, infertility, cancer, metabolic dysfunction, immunity and nervous system disorders [11]. Currently, highly selective PDE5 inhibitors are available as therapies to treat different human diseases [3]. A few examples include selective inhibitors of PDE3, PDE4 and PDE5 which were the earliest drugs approved by FDA for the treatment of some cardiovascular diseases (CVDs), i.e., hypertension, thrombosis and thrombosis associated-complications and congestive heart failure (CHF), whereas inhibitors of PDE2, PDE3, PDE4 and PDE10 have been exploited for neurodevelopmental disorders [2,3].

Considering PDE5 inhibitors’ (PDE5is) potent vasoactive effects, which result in vasodilatation, PDE5is are now licensed for the treatment of ED and pulmonary artery hypertension (PAH). In addition to these effects on the endothelium, PDE5is retain the ability to control the growth/division/death of cells and, remarkably, display powerful anti-inflammatory features. The latter aspect seems to be particularly relevant, considering that chronic inflammation is acknowledged as the common link between most diseases. The clinical exploitation of PDE5is may find a larger field of application in a wide-range of inflammation-based human diseases, beyond ED [12].

In this scenario, neuroinflammatory diseases represent a growing area of investigation and PDE5i exploitation. The aim of this review is to outline the state of the art and the potentiality of PDE5i as therapeutic tools to control neuroinflammation and, consequently neurodegenerative disorders in humans. Some of the main signaling processes involved in neuroinflammation and neurodegeneration will be addressed as potential therapeutic targets of PDE5is, focusing on sildenafil, the most promising molecule for neurological disease treatment.

## 2. PDE5 and Its Specific Inhibitors

PDE5 is a metallo-hydrolase that catalyzes the conversion of cGMP to 5′ GMP and controls different physiological activities within the body [13]. The PDE5A gene is located on the human chromosome 4q26, contains 23 exons (approximately 100 kilobases), encoding three alternatively spliced coding variants (PDE5A1-3), with different isoform-specific first exons, driven by specific promoters in response to cGMP or cAMP stimulation [14]. PDE5’s dimeric structure consists of two domains, GAF-A and GAF-B, which in concert control PDE5 dimerization [15]. The cGMP binding sites, responsible for PDE5 affinity to cGMP, are expressed in the GAF-A domain [13]. Following allosteric binding, cGMP is converted into inactive 5′ GMP.

PDE5 is almost ubiquitously localized in human tissues, including visceral and vascular smooth muscle, corpora cavernosa, skeletal muscle, platelets, lungs, the brain, spinal cord, kidneys, gastrointestinal tissue, prostate, bladder and urethra [14,16,17]. PDE5A1 and PDE5A2 are expressed in almost all human tissues, but PDE5A3 expression is present in tissues with a smooth and cardiac muscle component [16].

The three PDE5A isoforms share the same cGMP-catalytic activities, expressing variations only in the N-terminal region, and are similarly inhibited by sildenafil [18]. As previously addressed, cGMP is the primary target of PDE5; this second messenger engages in many critical downstream effects, including the regulation of calcium homeostasis, vasodilation, retinal phototransduction and neurotransmission. cGMP synthesis is under the control of the neurotransmitter nitric oxide (NO) through the activation of intracellular soluble guanylyl cyclase (sGC), that, in turn, triggers the activity of downstream cGMP-dependent protein kinases G (PKG) and ion channels, leading to a cascade of signals involved in important physiological effects [19]. Since cGMP accumulation is acknowledged as an inhibitory signal in inflammation, it is undeniable that this process represents a potential tool to limit and control the initiation/development of several inflammation-related diseases [20,21,22,23]. Chronic inflammation, also known as meta inflammation, is the leading cause of several pathologies, like metabolic and cardiovascular diseases, cancer and autoimmune and neurodegenerative disorders [24].

Concerning neurodegeneration, immune and neuroinflammatory mechanisms within nervous cells are well documented to trigger/contribute to disease pathogenesis, by secreting and engaging a variety of inflammatory mediators, i.e., cytokines, chemokines, and inflammation-related signaling paths [25]. That said, it is undeniable that PDE5 inhibition and, consequently, cGMP level stabilization are considered potential therapeutic interventions in neurological disorders [26]. Thus, due to the critical interference of PDE5 with cGMP signaling, PDE5-induced cGMP targeting was proposed for the treatment of several biomedical conditions, including neurological disorders, PAH, hypertension, cardiomyopathy, cancer, ED, and lower urinary tract syndrome [27].

## 3. PDE5, PDE5i and Neuroinflammation

Chronic inflammation is a hallmark shared by a large group of neurodegenerative disorders, including Alzheimer’s disease (AD), Parkinson’s disease (PD), amyotrophic lateral sclerosis (ALS), frontotemporal dementia (FTD) and Huntington’s disease (HD). Inflammation might affect different areas of the central nervous system (CNS) involving specific neuron subsets and different protein aggregates. It can be triggered by a variety of noxious stimuli, i.e., toxic metabolites, head injuries, infections, or autoimmunity, and plays a pivotal role in the progression of neurodegenerative disorders [28]. In physiological conditions, the blood–brain barrier (BBB), the highly specialized brain endothelium, maintains the neuronal microenvironment as optimally as possible to ensure the proper functioning of synaptic transmission and remodeling, neuronal circuits, neurogenesis and angiogenesis [29]. In physiological conditions, immune/inflammatory cells cross BBB with specific biomolecular interactions at very slow rate, upon inflammation the traffic of immunocytes, such as monocytes and T lymphocytes, and inflammatory mediators, i.e., cytokines like tumor necrosis factor (TNF)α and IL1-β, increases and triggers BBB impairments, perpetuating a vicious circle that allows neurodegeneration [30,31].

Growing evidence from clinical trials and experimental investigations shows that the deregulation of the signaling pathway between nitric oxide (NO), cGMP and protein kinase G (PKG) is tightly linked to neuroinflammation and neurodegeneration [32]. Indeed, cGMP-dependent signaling plays important physiological roles in the CNS, participating in neuronal survival, synaptic and cognitive function and the consolidation of memory [33]. I.e., cGMP stabilized levels through PDE5 inhibition, ameliorate neurogenesis via phosphoinositide 3-kinase (PI3K)/Akt pathway [34,35,36], which is engaged in synaptic plasticity. Interestingly, the NO-induced stimulation of the soluble guanylate cyclase (sGC)–cGMP-PKG pathway activates Akt and cyclic AMP-responsive element-binding protein (CREB); both are signal-transducers of neurotrophins aimed to neuroprotection [37,38]. In animal models, higher cGMP levels downregulate pro-apoptotic signaling in favor of the expression of anti-inflammatory signaling [39]. In progenitor cells of the retinal neuroglial, NO/cGMP/PKG retains antiapoptotic activity via Akt-CREB activation [40,41]. Moreover, there is some evidence for the role of a PDE5/cGMP-dependent cascade in the development of anxiety and depression, since cGMP levels are associated with inflammation, oxidative stress, impaired resilience to stressors and neuroplasticity, all factors contributing to neurological disorders [42,43].

Thus far, as the amount of second messengers within the cells is tightly regulated, ranging from nanomolar to millimolar concentrations, the perturbance and dysregulation of their production can lead to the dysfunction of or a disease within specific organs. In the CNS, several types of cells, including microglia, astrocytes, oligodendrocytes are under the regulation of PDE5-related signaling, as previously reported [28]. In this scenario, considering that the human brain expresses PDE5, the use of PDE5is to control cGMP levels retains the potential to be a therapeutic strategy against neuroinflammation and, therefore, against neurodegeneration [28,44].

Among the selective PDE5is approved by the US Food and Drug Administration (FDA) and by the European Medicines Agency, sildenafil, vardenafil, tadalafil and avanafil, only sildenafil and tadalafil can directly cross the BBB (some indirect evidence exists for vardenafil) [FDA, 1998. Viagra tablets (sildenafil citrate)] [45]. Indeed, sildenafil has emerged as being promising for the treatment of neurological diseases, likely due to its improvement of both vasculature and synaptic plasticity and neurogenesis [46,47]. Remarkably, besides improvements in blood flow and angiogenesis, sildenafil can increase the number of new synaptic connections and improve neurogenesis through cGMP/CREB, while decreasing apoptosis; it can counteract the formation of protein aggregates involving Akt and calpain/p25/cyclin-dependent kinase 5 (CDK5) pathways and hinder pro-inflammatory processes by decreasing pro-inflammatory cytokines [28]. Furthermore, PDE5i-induced increases in cGMP seem to reverse or restore the pathological cognitive signs of neurological diseases in part, i.e., AD and HD [48,49]. Sildenafil can counteract the formation of amyloid β (Aβ) plaques and protein aggregation increasing Akt, which, upon phosphorylation, inhibits GSK3β, which is involved in Aβ plaque deposition [50]. Interestingly, the decrease in proteins like glycogen synthase kinase 3 beta (GSK3β) or CDK5, the most relevant kinases engaged in Alzheimer’s disease pathogenesis, reduces the phosphorylation of the microtubule-binding protein tau, likely contributing to cognitive function restoration [51,52]. As has been found from studies in experimental autoimmune encephalomyelitis (EAE) models resembling multiple sclerosis (MS), sildenafil can reduce disease-associated clinical symptoms in association with a decrease in the levels of inflammatory cytokines, such as IL-1β, TNFα, and IL-17, involved in neuroinflammation and disease pathogenesis, such as IL-1β, TNFα, and IL-17, likely through the direct targeting of nuclear factor kB (NFkB) [20]. The overall effect induced by sildenafil that emerged in the MS experimental models can be described as a significant downregulation of the subset T helpers, (Th)1 (CD4+), which are critical for disease initiation and maintenance, and Th17, which are the main cells responsible for persistent inflammation and are the source of IL-17 which is highly expressed in blood, cerebrospinal fluid and local CNS lesions in MS patients [53,54,55,56,57]. To date, sildenafil can act against neuroinflammation by regulating the subset of Treg cells (Foxp3+) that limits the immune over response, by upregulating the expression of inhibitory cytokines, such as TGF-β and IL-10, and enhancing myelinization and synaptic plasticity through the brain-derived neurotrophic factor (BDNF) that is essential for neuron survival and cytoskeletal rearrangement [58].

## 4. PDE5 and PDE5i in the Establishment and Progression of Neurodegenerative Diseases

Intracellular signaling cascades of cyclic nucleotides are involved in multiple essential functions, including neuron specification and polarization as well as the establishment of the neuronal circuitries required for synaptic plasticity and for the accomplishment of neuromuscular and cognitive functions [59]. Impairments in these processes contribute to the development of several neurodegenerative diseases, including AD, HD, and PD.

**Figure 1:**
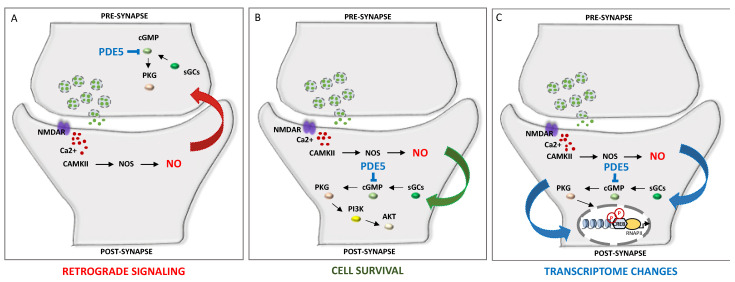
PDE inhibition contributes to cell survival and memory improvement via the modulation of NO signaling. NO, produced by NOS, leads to the activation of sGC and production of cGMP, which in turn activates PKG [63,64]. (**A**). Retrograde signaling of NO can promote the release of vesicles containing neurotransmitters in presynaptic neurons. (**B**). PKG also promotes cell survival via the AKT pathway. (**C**). PKG activation leads to CREB phosphorylation and the expression of memory-related genes. CaMKII: Ca2+/calmodulin-dependent protein kinase II; cGMP: cyclic guanosine monophosphate; CREB: cAMP response element-binding element; NMDAR: N-methyl-D-aspartate receptor; NO: nitric oxide; NOS: nitric oxide synthase; PI3k: phosphatidylinositol 3-kinase; PDE: phosphodiesterase; PKG: protein kinase G; sGC: soluble guanylate cyclase.

A fine-tuned regulation of cyclic nucleotides synthesis and hydrolysis, mediated by temporal and spatial features and achieved by more than 40 PDE isoforms, with different expression patterns and localization, offers a wide range of combinations of output signals to, in turn, adapt the specificity of response to distinct stimuli, shaping neuronal complexity and plasticity [60]. Thus, distinct PDE intracellular localization, kinetics and regulatory mechanisms enable the translation of a wide range of signals. As mentioned, subcellular compartmentalization of PDE enzymes represents a key step towards simultaneously generating multiple and contiguous cyclic nucleotides messages, shaping cellular microdomains. An additional layer of complexity is ensured by local interactions between cAMP and cGMP, which determine neurite maturation into axon or dendrite. The local ratio between cAMP and cGMP contributes to axonogenesis (with higher cAMP and lower cGMP) and dentritogenesis (with higher cGMP and lower cAMP). In developing neurons, the choice of the expression of excitatory or inhibitory neurotransmitter is modulated by the frequency of calcium spikes, which in turn are guided by cyclic nucleotides transients and by the activity of a set of effector kinases [61]. Hence, cAMP transients and calcium spikes are interdependent. In this way, the spatiotemporal dynamics of cyclic nucleotides determine, in time and space, neuronal polarization and neurotransmitter specification [62]. The dynamic function of PDE could also be achieved through its peculiar localization, i.e., the PDE5 protein is highly expressed in the cytoplasm of neuronal cells in human brains [44]; however, in the cortex and hippocampus, PDE5 is mostly expressed in pyramidal neurons, whereas in the cerebellum, it is prominently expressed in Purkinje neurons [44]. Figure 1 depicts the main mechanisms involved in PDE5-induced signaling cascade within neuronal cells.

In the following paragraphs we will describe and discuss the involvement of PDE5 activity in the onset and progression of neurodegenerative diseases, opening the path towards the development of PDEi as a valuable therapeutic opportunity.

### 4.1. PDE5i in Alzheimer’s Disease

AD is the most common form of dementia among the elderly. In AD patients, memory loss is accompanied by the formation of beta-amyloid plaques and the appearance of neurofibrillary tangles (NFTs) formed by hyperphosphorylated tau fibrils, which hamper proper neuronal functioning [65]. To date, several therapeutic approaches have been developed, including drugs inhibiting acetylcholinesterase (AChE) or antagonizing N-methyl-D-aspartate (NMDA), in addition to therapies aimed at inhibiting NFTs formation, interfering with tau protein function, thus decreasing Aβ load in the brain, inflammation and oxidative damage.

The failure of these strategies led researchers to focus on other non-amyloid-based approaches to restore memory function. In this context, promising candidates include PDEs. Interestingly, specific PDEis can improve memory performance in different AD animal models. In particular, the ability of PDE5i to interfere with the NO/cGMP/PKG/CREB signaling pathway by increasing the levels of cGMP has prompted the hypothesis that PDE5 inhibition might represent an effective therapeutic approach for the treatment of AD. Accordingly, the cGMP signaling path has been documented to play a pivotal role in cognition and memory function and represented a primary target in a trial using PDEi [66,67,68].

NO is a small gaseous molecule produced by the nitric oxide synthase (NOS) enzyme in L-arginine metabolism. NO can diffuse through cell membranes and participates in a wide range of physiological functions, including vasodilation, inflammation, neuroprotection, neurotoxicity and synaptic transmission. In the central nervous system (CNS), NO works as a neurotransmitter, going from the post-synaptic to the pre-synaptic neuron as a retrograde messenger. As mentioned above, the binding of NO to sGC stimulates the production of the second messenger cGMP, which activates its downstream effector PKG and the transcription factor cAMP-response element-binding element (CREB), thus promoting neurotransmission, synaptic plasticity and memory formation [69]. PKG also mediates neuroprotection via the inhibition of apoptosis throughout activation of the PI3K/Akt signaling pathway [70]. NO can also trigger the release of neurotransmitters, such as glutamate, at pre-synaptic neurons via activation of the sGC/cGMP/PKG pathway [71] (Figure 1). Furthermore, by achieving the S-nitrosylation of nuclear proteins, NO can also promote the binding of CREB to the DNA of target genes [38]. Upon nitrosylation, caspase enzymes and the N-methyl-D-aspartate receptor (NMDAR) reduce their activity, thus contributing to neuroprotection, whereas excessive NMDAR activity leads to abnormal intracellular Ca^2+^ levels and excitotoxicity [72].

Consistent with its neuroprotective function, the activation of NO signaling ameliorates altered neuroplasticity and memory deficits in animal models of AD [73]. Furthermore, in AD brains, a decreased phosphorylation level of CREB is observed, in line with the formation of Aβ and tau oligomers [63]. Notably, the reduction in pCREB phosphroylation correlates with reduced neuronal plasticity and memory formation, corroborating the link between impaired memory and NO signaling. In line with these observations, activation of the NO/sGC/cGMP/PKG/CREB pathway is sufficient to rescue Aβ or tau pathology and restore pCREB levels [46,73]. Remarkably, PDEis reproduce this pathway, ameliorating memory deficits.

The PDE5i zaprinastat, which also targets PDE6, PDE9 and PDE11, was the first to show improvements in cognitive functions in animals [74]. Sildenafil also showed improvements in recognition and spatial memory in both mice and rats [75,76,77]. Sildenafil treatment was shown to restore CREB phosphorylation in aged mice. Chronic administration of sildenafil in a 3-month-old transgenic APP/PS1 mouse model of AD could prevent cognitive deficits and synaptic dysfunction, at least in part by modulating the activity of CREB [50]. Interestingly, sildenafil treatment also diminished hippocampal Aβ levels. In addition, the administration of sildenafil to hippocampal slices reversed the impairment of LTP in the APP/PS1 mice [50,52]. Similarly, promising results were obtained in monkeys, clearly demonstrating that sildenafil can improve cognitive function in a dose dependent fashion [78].

Analogous results were obtained using other PDE5i, such as vardenafil and tadalafil [45,50,79]. Acute treatment with vardenafil was shown to improve spatial memory [80] and long-term memory performance [79]. Moreover, tadalafil was shown to reverse LTP reduction in slices of brain in an APP/PS1 mouse model of AD, but failed to achieve behavioral benefits when administered in vivo, probably due its poor BBB penetration [50,81]. Most recently, other PDE5is, such as icariin, yonkenafil, compound **7a** and compound **6c**, were developed specifically for their therapeutic potential in learning and memory. Icariin showed a strong beneficial effect against memory loss in APP/PS1 transgenic mice [82]. Treatment of APP/PS1 mice with yonkenafil improved working memory deficits. Moreover, yonkenafil was able to reduce the Aβ plaque area and to inhibit the over-activation of microglia and astrocytes, simultaneously increasing neurogenesis in the dentate gyrus [83]. Compound **7a** was shown to improve contextual and spatial memory in mice pre-treated with Aβ or tau oligomers, and in APP/PS1 mice. At the molecular level, increased cGMP and pCREB levels were observed in the hippocampi of AD mice [73,84]. By optimizing compound **7a**, the compound **6c** was developed, which demonstrated an ability to restore learning capacity and memory in the APP/PS1 transgenic mice [85] as well as improvement of synaptic plasticity in hippocampal slices. The in vivo administration of compound **6c** demonstrated positive behavioral outcomes [85]. These pre-clinical studies have been summarized in Table 1.

Several studies document that the memory improvement obtained with PDEis [50] was not related to their effects on blood flow [86]; consistently, intracerebroventricular administration of vardenafil was sufficient to ameliorate memory functions [87]. Nevertheless, it cannot be excluded that PDEi might enhance cognition also through vascular mechanisms [45].

Altogether, these reported findings strongly suggest the possibility of using PDE5is to enhance normal memory and age-related memory decline. However, despite the positive results obtained in pre-clinical protocols, the memory improvements from PDE5is observed in animal studies were not translated into clinical settings [88]. To date, none of the investigated drugs have reached the market for AD treatment. In fact, PDEi administration in humans has shown a wide variety of results, ranging from no effect to a beneficial effect on normal memory. Sildenafil administration to healthy volunteers [89] showed no significant effects on short-term memory [90], although it was demonstrated to enhance the ability to focus attention, select relevant target stimuli and improve information processing [90]. The NCT01940952 clinical trial was proposed to determine whether Zydena (Udenafil) had a positive effect on cognitive function in patients with AD (Table 2). The results obtained have not been disclosed yet.

### 4.2. PDE5i in Parkinson’s Disease

PD is a progressive disorder that occurs in later life, defined clinically by motor features and pathologically by neuronal degeneration and intraneuronal misfolded α-synuclein (Lewy bodies) in specific central and peripheral nervous system regions, including dopaminergic brain-stem neurons [91]. PD patients exhibit classic motor symptoms (e.g., asymmetric bradykinesia, rigidity, tremor, and imbalance) and cognitive deficits, particularly in the later stages of the disease [91].

To date, no therapy has been proved to slow disease progression. Although dopaminergic therapies can improve motor function, a loss of efficacy is frequently observed and several side effects have been documented [91]. However, regular exercise, a healthy diet, high-quality sleep and avoidance of adverse exposures have been associated with reduced mortality [92].

Interestingly, a reduced risk of PD was correlated with a high consumption of caffeine, which is able to increase cAMP and cGMP by inhibiting PDEs [93], suggesting increased PDE activity as a driver of PD. Nevertheless, neither mutations or reduced expression of PDE4D [94], PDE8B [95] or PDE10A [96] were shown in PD patients.

Remarkably, increased cyclic nucleotide signaling was shown in PD patients after pharmacological and electrophysiological therapies. Deep brain stimulation (DBS) of the subthalamic nucleus (STN), which is currently used to treat PD patients, transiently increases cGMP signaling in the striatum [97,98]. Furthermore, a reduced NOS expression in the striatum of PD patients was documented, potentially contributing to the decrease in cGMP production via sGCs [99].

An increase in neuronal NOS expression and activity was observed in PD patients, leading to an overproduction of NO and increased levels of cGMP from GC activation [100]. L-DOPA therapy, which can restore dopamine levels in PD patients, also increases cGMP levels in the serum and CSF of PD patients [100,101].

Animal models of PD can be created by using 6-hydroxydopamine (6-OHDA) or 1-methyl-4-phenyl-1,2,3,6-tetrahydropyridine (MPTP) to achieve lesions in dopaminergic neurons in the substantia nigra, which reduce cGMP in the striatum and globus pallidus (GP), and increase cAMP in the striatum [102,103,104]. Reduced NOS activity [104] leads to lower levels of NO and a reduced activation of sGC. However, the downregulation of cGMP by 6-OHDA may account for the increase in PDE1B expression [103,104], whereas the increase in cAMP could be due to the downregulation of PDE10A expression [103], which also occurs in PD patients [96]. Notably, different results were obtained using the two models: while 6-OHDA reduced NOS activity, MPTP treatment enhanced NOS expression and activity, thus increasing sGC, and, as a consequence, the cGMP levels in striatum and midbrain [105,106,107]. Importantly, MPTP-induced deficits were reversed by the PDE1 inhibitor vinpocetine [107,108] and by the PDE4 inhibitor rolipram [109].

Several clinical trials have been undertaken to evaluate the effectiveness of PDE5 inhibition as a therapeutic opportunity for the treatment of PD (Table 2). The NCT02162979 study is trying to determine whether sildenafil is effective in reducing dyskinesias in patients with PD, whereas the NCT01941732 trial is evaluating motor function and cerebral blood flow (CBF) in PD patients before and after sildenafil intake and before and after anti-PD medication, since a number of patients have reported that when they take sildenafil, their need for anti-PD medication is reduced. The rationale of this trial resides in the role of sildenafil in increasing brain blood flow, hence improving the function of specific brain regions, ameliorating motor function. Lastly, the purpose of the NCT02225548 study is to evaluate whether selegiline and tadalafil can improve erectile dysfunction (ED) in male patients with Parkinson’s disease (PD) and moderate ED. Selegiline acts as a monoamine oxidase inhibitor (MAOI) and thereby increases levels of monoamine neurotransmitters in the brain [110]. It is prescribed to PD patients that are taking carbidopa/levodopa who are not receiving the complete benefits of carbidopa/levodopa. Nevertheless, the results obtained from these studies have not been disclosed yet.

### 4.3. PDE5i in Huntington’s Disease

HD is an autosomal-dominant progressive neurodegenerative disorder associated with the expansion of CAG/polyglutamine repeats in the Huntingtin gene, primarily causing the degeneration of striatal neurons, and involving additional neuronal populations, such as cortical and hippocampal neurons. This scenario leads to depression and cognitive dysfunction, preceding the motor deficits [111].

Several reports document altered cAMP signaling in the striatum, hippocampus and cortex of HD patients and animal models of the disease [112]. This could be due to a reduced degradation of the PDE4 protein and a concomitant increase in PDE4 activity, driving the depression-like phenotypes, but not the motoric phenotypes, seen in HD mouse models [113].

Furthermore, HD mouse models also show reduced cGMP levels in the hippocampus, possibly due to a loss of nNOS signaling, that could contribute to the decrease in the NO-stimulated sGC activity [114]. Altered cAMP and cGMP levels drive the compensatory decreases in the expression of PDE10A and PDE1B [115,116], as documented in both HD patients [116,117,118] and animal models [115,116,119]. Remarkably, PDE10A inhibitors can reduce behavioral, neurodegenerative, and electrophysiological deficits in HD animal models [111,115,120], possibly via increasing pCREB in striatum, cortex and hippocampus in both HD patients and animal models [121].

Regarding PDE5, it was demonstrated that sildenafil and vardanafil had neuroprotective roles against the mycotoxin 3-nitropropionic acid (3-NP), and were able to induce behavioral and biochemical abnormalities by inhibiting the succinate dehydrogenase (SDH) activity, thus resulting in mitochondrial dysfunction and cellular energy deficits, and producing striatal lesions closely mimicking the neuropathological features of HD [122]. Both sildenafil and vardanafil, as well as the PDE4 inhibitor RO 20-1724, significantly attenuated 3-NP induced neurotoxicity [123]. The molecular mechanism relies on the inhibition of calpain activation, and on the increase in p-CREB and BDNF levels [124]. In line with these positive results, the PDE5is sildenafil and tadalafil were shown to reduce the levels of the mutant Huntingtin and tau proteins in zebrafish models of tauopathies or HD via the regulation of protein ubiquitination and overall protein degradation through PKG phosphorylation and activation [125]. These findings highlight the therapeutic potential of treatments which raise cGMP to counteract different proteotoxic diseases, including cardiac failure and ischemia [126], via the PKG’s ability to enhance global protein degradation [127]. Overall, the activation of PKG could represent a promising therapeutic strategy to counteract untreatable proteotoxic diseases.

## 5. Conclusions

There is growing evidence showing that neuroinflammation is a trigger for neurodegenerative disease initiation and progression, and not merely a consequence. Acting on inflammatory processes preceding neurological disease could be a useful approach to limit neurodegeneration. Different anti-inflammatory strategies failed to delay disease progression in clinical trials, likely due to the complex role of inflammatory signaling in neurodegenerative processes, displaying at the same time beneficial and detrimental effects [25]. Nevertheless, in this scenario, the inhibition of PDE5 activity emerges to retain its potential as a safe and beneficial strategy in neurological diseases. Sildenafil seems to be a promising approach, thanks to its multi-dimensional improvement on vasculature, synaptic plasticity and neurogenesis.

As previously addressed, sildenafil is the most studied and promising molecule due to its ability to cross the BBB. As seen in studies in mice and rats, tadalafil can ameliorate neurorepair and neurological performance (i.e., object recognition task), suggesting some central beneficial effects [128], despite its poor penetration into the BBB. Independently from the drug’s ability to cross BBB, many studies refer to the increase in CBF induced by PDE5is as potential treatment to ameliorate vascular cognitive impairment, characterized by reduced CBF. Remarkably, the middle cerebral artery’s flow velocity, which is mostly used to estimate CBF, has shown improved cerebral responsiveness after PDE5i treatment [128]. So far, it seems that the benefit likely relies on the improved responsiveness of the vasculature and, presumably, on some released mediators being able to cross the BBB.

It is mandatory to underline the paucity of research studies and the high variability that exists in the quality of data due to different protocols for intervention, route of administration or outcome measures. In addition, most of the studies are performed on animal models, and are not immediately ascribable to humans.

Previous studies on other diseases show that sildenafil can counteract inflammation by interfering with inflammatory molecules and signaling at either the local or systemic level, and this can be an undeniable advantage [129,130,131]; furthermore, it has been proven to be a safe drug licensed for ED and pulmonary hypertension.

Based on this observation, PDE5 inhibition could have clinical relevance in other diseases in which counteracting inflammatory and (auto)immune over response could be a promising therapeutic approach [12,132]. This is the case of PDE5i proposed as treatment options for cardiomyopathy or cancer (and also as sparing-agents) due to their off-target effect to interfere with aberrant signals [133,134,135,136,137]. For example, tadalafil has a label indication for the treatment of benign prostatic hyperplasia (BPH) [138]. It is noteworthy that PDE5is, in addition to their label indication for PAH in Systemic Sclerosis (SSc), are currently given off-label for pulmonary hypertension secondary to other rheumatologic diseases, and for Raynaud phenomenon or digital ulcers in SSc [139]. So far, PDE5is retain their potentiality as disease-modifying agents due to their broader spectrum of molecular interactions and multi-target effects.

To date, none of the PDE5 inhibitors is totally selective, and the cross-reactivity with other PDE isoenzymes is likely the reason for many of the negative effects caused by these drugs [140]. Indeed, PDE5i administration should be carefully evaluated since there are data reported on some non-negligible side effects, including melanoma, altered vision and optic neuropathy, altered blood pressure, prostate cancer, dyspepsia, back pain and myalgia, headaches, flushing, priapism, rhinitis and hearing loss [140].

In this scenario, it is undeniable that a more exhaustive and deeper understanding of the cellular/molecular mechanisms underlying neuroinflammation and neurodegeneration will be a step forward in individuating target(s) and subjects for the safe treatment of neurodegenerative diseases. Large-scale studies and trials are mandatory, as well as translational research in humans to support the PDE5is’ potential as treatments for neurological disorders, likely in the view of precision, personalized medicine.

## Figures and Tables

**Table 1:** Pre-clinical studies evaluating PDE5 inhibitors in neurodegenerative diseases.

PDE5 Inhibitor	Animal Models	Results	Reference
**Tadalafil**	3-month-old J20 transgenic mice	Improved memory performance	[45]
**Sildenafil**	Double transgenic mice expressing both the human APP and PS1 mutations compared with wild-type littermates	amelioration of synaptic function, CREB phosphorylation, and memory	[50]
**Zaprinast**	3-month-old Tryon–Maze–Bright rats	improved memory consolidation	[74]
**Sildenafil**	6-month-old Swiss mice	improved memory consolidation	[75]
**Sildenafil**	2-month-old Swiss mice	Improved performance	[77]
**Sildenafil**	Cynomolgus macaque	improved object retrieval performance	[78]
**Vardenafil**	4–5-month-old mice	Improved spatial memory acquisition and early consolidation	[80]
**Yonkenafil**	Double transgenic mice expressing both the human APP and PS1 mutations compared with wild-type littermates	Rescue of cognitive deficits and amelioration of amyloid burden	[83]
**Compound 7a**	Double transgenic mice expressing both the human APP and PS1 mutations compared with wild-type littermates	Increased level of cGMP in mouse hippocampus and amelioration in synaptic plasticity and memory	[84]
**Compound 6c**	Double transgenic mice expressing both the human APP and PS1 mutations compared with wild-type littermates	Increased level of cGMP in mouse hippocampus and amelioration in synaptic plasticity and memory	[85]

**Table 2:** Ongoing clinical trials evaluating PDE5 inhibitors in neurodegenerative diseases.

NCT Number	Study Title	Conditions	Interventions
NCT02162979	Sildenafil (Viagra) for the Treatment of Dyskinesias in Parkinson’s Disease	Parkinson’s Disease	Sildenafil, Placebo
NCT01940952	Zydena on Cognitive Function of Alzheimer’s Disease Patients	Alzheimer’s Disease	Zydena (Udenafil) + Donepezil, Placebo + Donepezil
NCT01941732	Motor Response to Sildenafil in PD	Parkinson’s Disease|Erectile Dysfunction	Sildenafil
NCT02225548	data Sagene 2014—Parkinson’s Disease and Erectile Dysfunction	Parkinson’s Disease|Erectile Dysfunction	Selegiline, Tadalafil
